# Design and validation of an immersive virtual rehabilitation system for individuals with vestibular disorders: A feasibility study

**DOI:** 10.1371/journal.pone.0354872

**Published:** 2026-07-30

**Authors:** Abdulaziz Ibrahem-Altwijri, Sergio Albiol-Pérez, María-Dolores Cortés-Vega, Cristina García-Muñoz, Rocio Sasián Ramírez de Arellano, José-Antonio Gil- Gómez, José-Antonio Lozano-Quilis, María Jesús Casuso-Holgado

**Affiliations:** 1 Systems Engineering and Computing, Universidad de Zaragoza, Zaragoza, Spain; 2 Aragón Health Research Institute (IIS Aragón), Universidad de Zaragoza, Zaragoza, Spain; 3 Departamento de Fisioterapia, Universidad de Sevilla, Sevilla, España; 4 Departamento Ciencias de la Salud y Biomédicas, Universidad Loyola Andalucía, Seville, Spain; 5 Unidad de vértigo y equilibrio, Grupo Recoletas López Cano, Cádiz, Andalucía, Spain; 6 Instituto Universitario de Automática e Informática Industrial, Universitat Politècnica de València, Valencia, Spain; 7 Instituto de Biomedicina de Sevilla (IBiS), Departamento de Fisioterapia, Universidad de Sevilla, Sevilla, España; 8 CTS 1110: UMSS Research Group, Junta de Andalucía, Seville, Spain; UFPE: Universidade Federal de Pernambuco, BRAZIL

## Abstract

**Background:**

Vestibular rehabilitation is an essential part of managing balance disorders, but traditional exercises can often be repetitive and demotivating for patients. This can reduce adherence and limit treatment outcomes.

**Objective:**

This study aimed to assess the feasibility, usability, patient satisfaction, and safety of DizzyVR, an immersive virtual reality system designed to support vestibular rehabilitation. Preliminary data on its potential effects on clinical outcomes were also collected.

**Methods:**

A prospective, single-arm feasibility study was conducted with ten participants diagnosed with various vestibular disorders. Each participant completed eight weekly sessions using DizzyVR. Feasibility was assessed through session attendance and completion rates. Usability, satisfaction, and safety were measured using the System Usability Scale, the User Satisfaction Evaluation Questionnaire, and the Simulator Sickness Questionnaire. Clinical measures included the Dizziness Handicap Inventory (DHI), Timed Up and Go (TUG) test, Activities-specific Balance Confidence (ABC) scale, and Functional Gait Assessment (FGA).

**Results:**

Ten participants completed the training, with a high adherence rate (91.25%). Usability scores indicated good ease of use, and satisfaction scores were high. Mild adverse events such as nausea or disorientation were reported by three participants, but improved over time. Exploratory pre–post changes were observed in gait speed (TUG: p = 0.002), balance confidence (ABC: p = 0.007), and gait stability (FGA: p = 0.012). While DHI scores demonstrated a trend toward improvement, the change did not reach statistical significance (p = 0.081). Overall, participants reported feeling safe and expressed willingness to recommend and reuse the system.

**Conclusions:**

The findings suggest that DizzyVR is a feasible, usable, and well-accepted tool that may complement conventional vestibular rehabilitation. The system appears to support patient motivation and safe participation while showing promising exploratory changes in balance and gait. Further studies with larger samples, control groups, and longer follow-up are recommended to confirm and expand on these promising preliminary results.

## 1. Background

The vestibular system (VS) is a complex sensory network that involves communication between vestibular organs of the inner ear, the eyes, postural muscles, brainstem, cerebellum, cortex, and other vestibular central pathways [1]. This complex processing provides our brains with a deep understanding of how gravity affects our body, how the head is positioned in relation to our body, and how our body is positioned in space, allowing the brain to predict trajectories with startling accuracy, among other functions [2]. Normal function of the VS is crucial for posture and gait, ensuring that vestibulo-ocular reflex (VOR) and vestibulospinal reflex (VER) operate accurately, eye movements occur with very short latencies, and muscle tone is adjusted accordingly [3].

Vestibular disorders are relatively common [4] and can be caused by various peripheral or central mechanisms, ranging from head and neck trauma [5] to neurological diseases such as multiple sclerosis [6] and vestibular migraine [7]. According to [8] Strupp et al., about 2.7% of adults in the USA have these disorders. Benign paroxysmal positional vertigo, Ménière’s disease, vestibular migraine, and neuritis are among the most prevalent vestibular conditions [8]. When vestibular disorders occur, the main symptoms are dizziness, as well as altered perception of body position and motion, ocular motor control, posture, gait, and balance [8,9].

Research has shown that vestibular rehabilitation programs, including gaze fixation, postural control, and balance exercise, can significantly reduce dizziness and improve balance and gait in adults [10]. Vestibular rehabilitation focuses on retraining the vestibular sensory system through repetitive exercises in different positions [11]. These exercises focus on stimulating the VOR and cervical-ocular reflexes [1], retraining somatosensory information, balance, and gait [2], and are based on the physiological mechanisms of adaptation, habituation, and substitution. [7]. Regarding vestibular rehabilitation programs, Cawthorne and Cooksey (1946) were pioneers in the therapeutic approach to vestibular dysfunctions, proposing what is still considered the gold-standard protocol for vestibular rehabilitation [12]. This protocol consists of an exercise plan designed in increasing order of difficulty, structured into five levels: 1) sitting with eye and head movements; 2) sitting with head and body movements; 3) standing exercises; 4) dynamic balance and walking; and 5) adding visual restriction to levels 3 and 4.

Game-based virtual reality (VR) systems have been increasingly used in rehabilitation settings and have shown potential benefits for patients with vestibular disorders [13]. These systems help improve the key processes involved in vestibular therapy: habituation, by exposing patients to repeated movements in a controlled environment; adaptation, by providing visual feedback to help retrain eye and head coordination; and substitution, by offering other sensory cues to help the brain rely on alternative pathways when balance is affected [13]. VR systems designed as games have been increasingly applied in the rehabilitation environment and have demonstrated the potential to help patients with vestibular disorders [13].

VR systems have been developed to provide vestibular rehabilitation, though most have used general environments, commercial software, or rudimentary optokinetic stimulation rather than exercises tailored to the vestibular system. Although these systems have been shown to improve balance, gait, and dizziness in other studies [14,15], they are associated with significant limitations: they tend to provide less task-related training associated with Activities of Daily Living (ADL), provide fewer choices regarding individual progression, and lack objective kinematic monitoring. These factual lacunae point to more specialized methods. DizzyVR was to deal with these limitations and offer a block-based protocol which was based on the Cawthorne Cooksey framework, immersive exercises that work on gaze, postural control, coordination, and gait, and in-built progression criteria and objective performance recording. Unlike their system, our immersive virtual reality (IVR) approach offers task-specific rehabilitation tailored to daily activities, with extended usability, objective performance tracking, and higher potential for long-term functional gains [15].

Existing VR-based systems have limitations such as low adherence and a lack of real-time feedback. Therefore, it is necessary to test a fully customizable IVR. The Dizzy Virtual Rehabilitation (DizzyVR) is designed for patients with vestibular system dysfunctions, thanks to the recommendations from clinical specialists and participant experiences. This system is based on the gold-standard vestibular rehabilitation protocol proposed by Cawthorne and Cooksey (1946), aiming to emulate gaze fixation, oculomotor coordination, posture control, and gait through its exercises.

This study aims to evaluate the feasibility, usability, patient satisfaction, and safety of the immersive virtual reality system denominated DizzyVR. This study also explores preliminary clinical outcomes related to the impact of this new vestibular rehabilitation system on patient performance and balance-related measures.

## 2. Materials and methods

### 2.1. Study design

This is a mixed-methods, prospective, single-arm feasibility study. The study protocol was approved by the Coordinating Committee for Ethics in Human Research of Andalucia (CCEIBA 1/12/2023) and registered at ClinicalTrials.gov (NCT06350721). The study was performed in accordance with the Declaration of Helsinki. The individual presented in Fig 6 has provided written informed consent for publication of the image in accordance with PLOS consent requirements.

### 2.2. Settings and participants

The participants were contacted and recruited in the Otorhinolaryngology Department of Dr. López Cano Hospital in Spain. The therapist recruited a convenience sample of ten participants. This sample size is like previous feasibility studies about this topic [16].

The inclusion criteria are the following: 1) Man or woman between 18 and 75 years old; 2) patients with a confirmed diagnosis of central or peripheral vestibulopathy; 3) ability to walk; and 4) presence of dizziness symptoms as assessed by the Dizziness Handicap Inventory (> 10 points) [17].

The exclusion criteria are the following: 1) Severe visual disturbances; 2) cognitive impairment, with a Mini-Mental State Examination score < 24 [18]; 3) comorbidity that affects postural control and balance severely; and 4) uncontrolled systemic diseases that contraindicate physical activity.

### 2.3. The DizzyVR system

DizzyVR includes a broad set of activities designed to achieve specific rehabilitation goals. The training is divided into distinct blocks, each adapted to a specific exercise. Block 1, for example, is concerned with gaze fixation and is broken into three sections: moving the eyes without moving the head, moving the head without moving the eyes, and coordinating the movement of both the eyes and the head. Postural control (Block 2), eye-hand coordination (Block 3), gait (Block 4), and visual input restrictions (Block 5) are covered in the following blocks.

These are unique workouts that, in many cases, can overcome the challenges associated with vestibular imbalance or dysfunction. The goal of these workouts is to develop a tolerance mechanism, and the more carefully and consistently they are performed, the sooner the symptoms will subside.

The DizzyVR system was developed using the Unity engine and implemented using the VIVE OpenXR PC VR plugin. The system was deployed using a head-mounted display (HTC Vive Pro 2), two handheld controllers, and external base stations for room-scale tracking. A SensingTex Kit was also used to support balance-related interactions.

The system records head and hand position and rotation, as well as object-related data within the virtual environment. Tracking data are recorded once per second, including spatial coordinates and rotation values. Session data such as completion time and performance scores are also recorded.

The VR setup requires a room-scale environment with a minimum play area of approximately 2 × 1.5 meters, with base stations positioned between 2 and 2.5 meters in height. The system runs on a PC with at least an Intel i5 processor (or equivalent), NVIDIA GTX 1060 (or higher), 4 GB RAM, and Windows 10 operating system. The technical specifications of the DizzyVR system are summarized in Table 1.

**Table 1:** Technical specifications of the DizzyVR system.

Component	Specification
Head-mounted display	HTC Vive Pro 2
Tracking system	Head and hand position and rotation tracking
Controllers	Two handheld controllers
Additional sensors	SensingTex Kit
Software platform	Unity + OpenXR
Tracking frequency	1 Hz
Play area	2 × 1.5 m
Base stations height	2–2.5 m
Minimum PC	i5, GTX 1060, ≥ 4GB RAM

**Fig 1:**
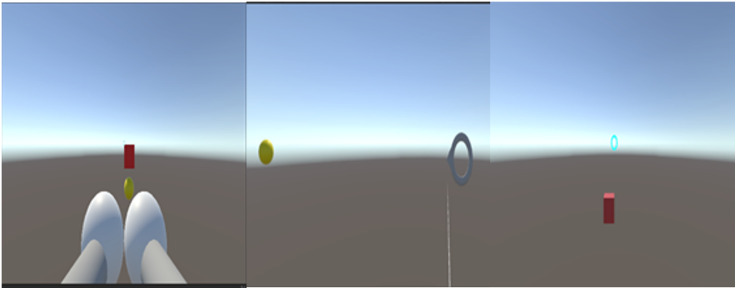
Gaze Fixation Training in Block 1 of the DizzyVR Protocol (original content developed by the authors).

DizzyVR is divided into blocks based on distinct movements. The therapist can customize each exercise level. The activities assigned in each block are intended to be simple for participants. In Block 1 (gaze), for example, users focus their sight on “the cube” while tracking “the sphere” with their eyes in the “Moving the eyes without moving the head” activity. Participants use a hand pointer to point at the sphere to guarantee accurate eye tracking (see Fig 1).

In “Moving the head without moving the eyes,” participants are shown a compass in the center of the screen, leading to a hidden treasure. The goal is to keep steady eye contact with the compass and shift the head in the direction of the arrow. Once the treasure has been found, the compass will take the user to the next one. For “To move the eyes and the head,” participants are advised to synchronize the movement of their head and eyes as they follow “the cube,” as seen in the following illustrations.

**Fig 2:**
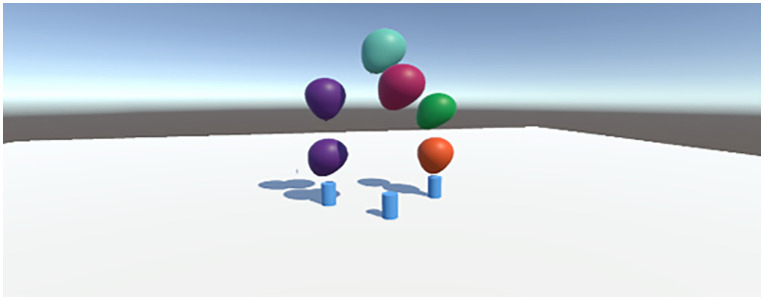
Postural Control Training in Block 1 of the DizzyVR Protocol (original content developed by the authors).

Block 2 focuses on postural control by having participants rise up and sit down while including head motions in various directions to identify and interact with balloons (see Fig 2).

**Fig 3:**
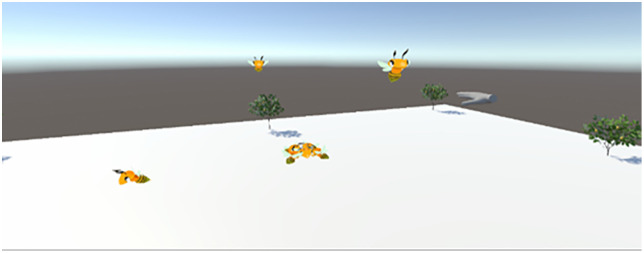
Illustration of Block 3 (Eye–Hand Coordination) from the DizzyVR vestibular rehabilitation program (original content developed by the authors).

Moving on to Block 3, which focuses on eye-hand coordination, participants complete a challenge in which they must successfully coordinate the motions of their eyes and hands to lead bees back to their lemon trees. Bees emerge unexpectedly, and a gratifying clap sound indicates that the contact was successful as the bee returns to its tree. This exercise can be done with one or both hands (see Fig 3).

**Fig 4:**
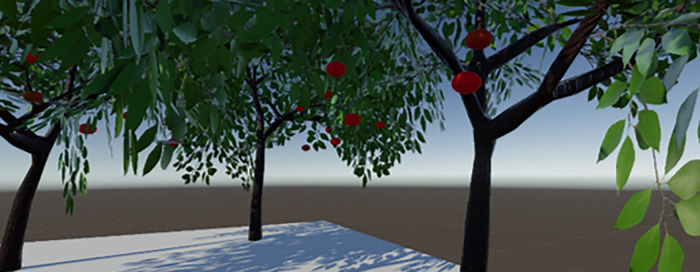
Illustration of Block 4 (Gait) from the DizzyVR vestibular rehabilitation program (original content developed by the authors).

Block 4 focuses on gait; participants walk through the exercise while concurrently changing their head movements to avoid branches. The goal is to discover apples and capture them with accurate hand movements (see Fig 4).

**Fig 5:**
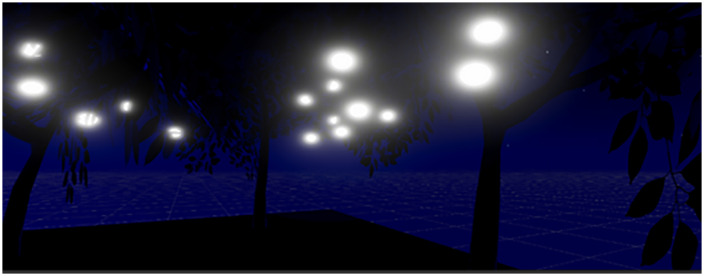
Overview of the low-light visual input restriction task (Block 5) designed to enhance gaze and balance control in the DizzyVR rehabilitation system (original content developed by the authors).

Block 5 concludes with a visual input restriction exercise that is identical to the previous one but uses lower illumination to mimic restricted visual input. Participants are tasked with capturing fireflies before they fly away, which increases the difficulty of the task. Because of the reduced illumination, users must rely on their visual acuity and agility to effectively catch the firefly before it changes positions (see Fig 5).

We have used the HTC Vive 2 headset for this exercise. The breakdown of the DizzyVR system’s exercises, along with instructions, is presented in Table 2.

**Table 2:** Summary of DizzyVR training blocks, exercise descriptions, and configuration parameters.

Block	Exercise	Instructions	Configuration
Block1: Gaze Fixation	Move eyes without moving the head.	Focus on the cube while tracking a moving sphere with only the eyes. Use a hand pointer for precise tracking.	Time: 1–60 minutes. Speed: low, medium, and high scale. Amplitude: low, medium, and high scale. Directions: vertical, horizontal, diagonal, zigzag, and star shape. The color gaze: multiple possibilities.
	Moving head without moving eyes.	Keep eyes fixed on a central compass while moving the head in the direction of the arrows to find hidden treasures.	Time: 1–60 minutes. Speed: low, medium, and high scale. Amplitude: low, medium, and high scale. Directions: vertical, horizontal, diagonal, zigzag, and star shape. Gaze color: multiple possibilities.
	Moving eyes and head together.	Synchronize head and eye movements to follow the cube across the screen.	Time: 1–60 minutes. Speed: low, medium, and high scale. Amplitude: low, medium, and high scale. Directions: vertical, horizontal, diagonal, zigzag, and star shape. Gaze color: multiple possibilities. A clapping sound when the user finishes the session.
Block 2: Postural Control	Sit-to-stand with head movement.	Perform standing and sitting movements while turning the head to spot and interact with balloons.	Time: 1–60 minutes. Speed: low, medium, and high scale. Area: Head level, down head, over head, and altogether. Scale: Big, medium, and small scale.
Block 3: Eye-Hand Coordination	Guiding bees to lemon trees.	Use one or both hands to point and guide bees back to their trees; a clap sound confirms success.	Time: 1–60 minutes. Speed: low, medium, and high scale. Area: Head level, down head, over head, and altogether. Scale: Big, medium, and small scale.
Block 4: Gait	Walking and apple collection.	Walk forward while avoiding branches by moving the head and accurately using hands to collect apples.	Time: 1–60 minutes. Speed: low, medium, and high scale. Area: Head level, down head, over head, and altogether. Scale: Big, medium, and small scale.
Block 5: Visual Input Restriction	Firefly capture in low light.	Catch fireflies in a dim environment, requiring quick, accurate hand-eye coordination under reduced lighting.	

The DizzyVR program’s user manual lists the precise settings and output data parameters for every block.

The setup for Block 1, which focuses on Gaze Fixation, provides options for gaze color, direction (vertical, horizontal, diagonal, zigzag, and star shape), amplitude (low, medium, and high), and time (1–60 minutes). In each of the five blocks, the output values were session parameters (time, score, session duration, and settings of the configuration) and detailed tracking data (e.g., the head, hand, and object positions and rotations measured every second).

Block 2, which is all about Postural Control, has the following configurations: area (head level, down head, overhead, and all together), scale (large, medium, and tiny), length (1–60 minutes), and speed (low, medium, and high). While session data contains time, score, length of session, and configuration parameters, output data includes tracking information for head position and balloon positions per second.

Block 3, which deals with Eyes-Hands Coordination, has four configurations: area (head level, down head, overhead, and all together), scale (large, medium, and small), and time (1–60 minutes). The tracking data includes bee locations, head, left, and right-hand positions, and rotation. Time, score, length of the session, and configuration details are all recorded in the session data.

Configurations for time (1–60 minutes), speed (low, medium, and high), area (head level, down head, overhead, and all together), and scale (large, medium, and small) are included in Block 4, which focuses on gait. The tracking data includes the positions of the head, left and right hands, rotation, and apples. Time, score, length of the session, and configuration details are all included in the session data.

In the output data portion, Block 5, Visual Input Restrictions, is finally explained. It includes tracking information that records the locations and rotations of the head, hand, and apple. Time, score, length of the session, and setup information are all included in the session data.

### 2.4. Outcome measures

The baseline characteristics of the patients, including demographic data (age, gender, ethnicity), body mass index, type and date of vestibular diagnosis, as well as experience with VR devices (yes/no), were collected.

**Fig 6:**
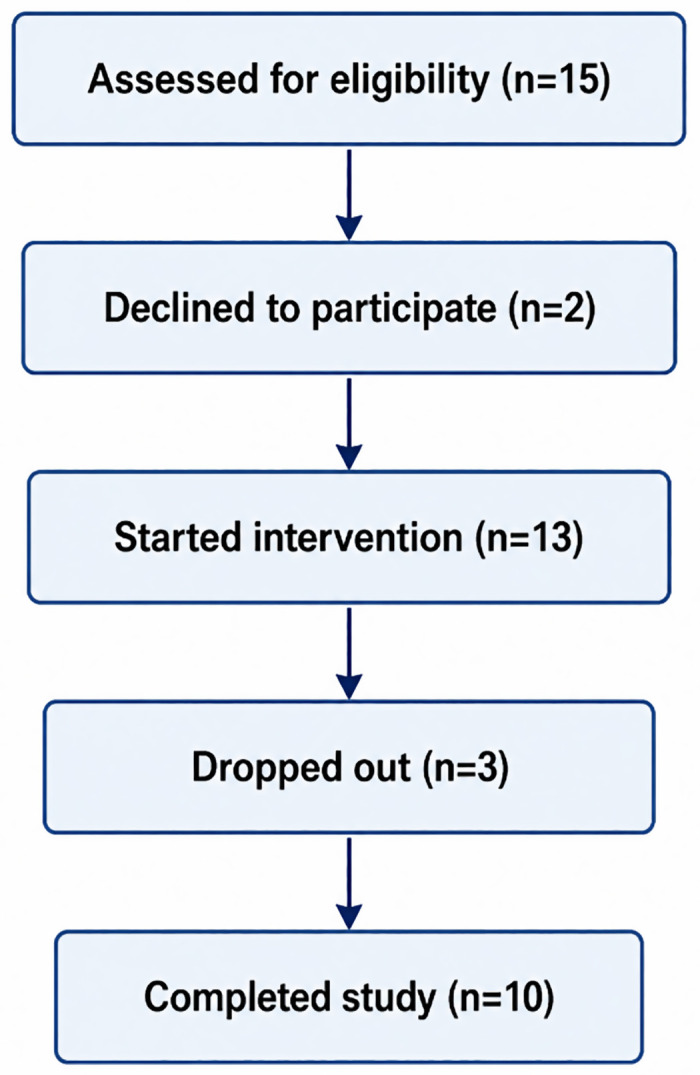
Flow diagram of participant recruitment and retention in the single-arm feasibility study.

The primary feasibility endpoint was protocol adherence, defined as the proportion of completed DizzyVR sessions out of the total planned sessions. A success threshold of ≥80% adherence was considered indicative of feasibility. Secondary outcomes included usability, patient satisfaction, and safety. Exploratory outcomes included changes in clinical measures (DHI, TUG, ABC, and FGA). Additional feasibility-related metrics included success rates in the games and progression through each level. The System Usability Scale (SUS) was used to assess usability [19], the User Satisfaction Evaluation Questionnaire (USEQ) to assess patient satisfaction [20], and the Simulator Sickness Questionnaire (SSQ) in combination with an adverse events registry to assess the safety of the system [21] (Fig 6).

The SUS questionnaire is composed of 10 questions related to usability in general terms, using a Likert scale from 1 to 5 (1 indicates that the item strongly disagrees and 5 indicates that it strongly agrees) [19].

The USEQ is a short, easy-to-use questionnaire designed to measure the user experience of people when they interact with technological systems, especially for people with motor impairments [20].

The SSQ is a Likert scale from 0 to 4, indicating the intensity of the feeling of the participant. This questionnaire is classified into three sub-categories: 1) Nausea (general discomfort, increased salivation, stomach awareness, burping, sweating, nausea, and difficulty concentrating); 2) Oculomotor stress (general discomfort, blurred vision, headache, eyestrain, fatigue, difficulty focusing, and difficulty concentrating); and 3) Disorientation (dizzy with eyes open, dizzy with eyes closed, head fullness, vertigo, blurred vision, nausea, and difficulty focusing) [21].

Although feasibility studies cannot address the effectiveness of an intervention, we collected preliminary data on exploratory clinical outcomes of the training intervention by assessing four secondary outcomes related to clinical symptoms. Dizziness was assessed using the Dizziness Handicap Inventory (DHI) [22]; gait speed using the Timed Up and Go test (TUG) [23]; balance confidence using the Activities-specific Balance Confidence Scale (ABC) [24]; and gait stability using the Functional Gait Assessment questionnaire (FGA) [25]. The DHI is a 25-item self-assessment questionnaire designed to measure the self-perceived handicapping effects caused by dizziness and balance problems [17].

The TUG was designed to obtain the time that patients used to stand up from a chair, walk a distance of 3 m, turn around, return, and sit down on the chair. This clinical test is related to dynamic balance [23].

The ABC is a questionnaire designed to measure how confident a person feels about not losing their balance during different ADL [24].

The FGA is a 10-item gait test based on the Dynamic Gait Index (DGI) that assesses postural stability during walking tasks, specifically adapted for people with vestibular disorders [25].

Finally, a semi-structured interview was conducted to assess the participants’ qualitative experiences of using the DizzyVR system. This comprised a series of open-ended questions covering usability, satisfaction, safety, and perceived clinical effect. Information was also collected on how the tool could be improved. Each interview was recorded in full on a voice recorder and transcribed verbatim. No computer software was necessary for data analysis; a system of categories and codes was constructed to organize and compare all the information (Table 3).

**Table 3:** Categories and coding framework used for qualitative analysis of participant interviews.

Categories	Codes
Usability/satisfaction	Simplicity of use Use of the device at home without supervision by a physical therapist Level of satisfaction Usefulness of the tool Suggestions for improvement
Safety	Safety VS Dizzying sensation Discomfort during treatment Discomfort is preventing further treatment
Perceived clinical effects	Improvement of symptoms Other treatments received Recommendation of treatment/repeat treatment

### 2.5. Study procedures and training intervention

Once the patients were admitted to the Physiotherapy Department of the Vertigo Unit, the hospital where the study was conducted, patients were given an information sheet and an informed consent form. The project was then explained to them. After signing the informed consent form, all descriptive variables were collected, as well as the baseline score on the DHI questionnaire, TUG, ABC scale, and FGA questionnaire (T0). Throughout the course of the study, participants’ attendance, the overall percentage of success in each game, the occurrence of adverse events, and the SSQ scores were recorded daily by the therapist (T1). At the end of the final assessment session (week 10), the DHI questionnaire, TUG, ABC scale, FGA questionnaire, as well as the SUS questionnaire and the USEQ scores were collected (T2). Finally, the semi-structured interview was conducted.

**Fig 7:**
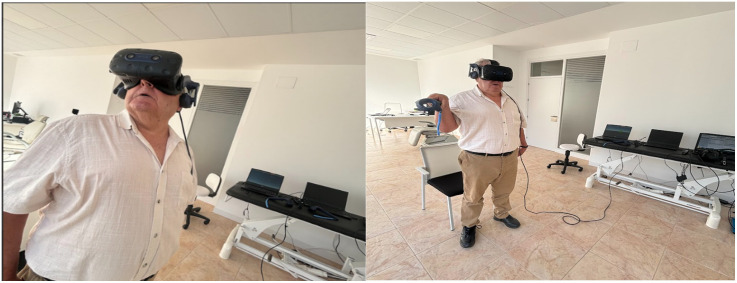
Participant performing a vestibular rehabilitation session using the DizzyVR system (Written informed consent for publication of the identifiable image was obtained from the participant).

Each patient received a total of eight sessions of vestibular rehabilitation using the DizzyVR system (see Fig 7). Each session lasted 50 minutes and was administered once a week. Seven different games were used to train to address different key functions of the vestibular system. The time period of each game was set at seven minutes. The intervention was executed on the premise that the physiotherapist would progress to a higher level if the patient demonstrated a successful completion rate of 80% or more [26]. The total duration of the intervention was 10 weeks, with the baseline assessment (T0) in the first week and the final assessment (T2) in the tenth week.

### 2.6. Statistical analysis

Data analysis was carried out using the Statistical Package for the Social Sciences (SPSS, version 30.0, IBM Corp., Armonk, NY, USA). The Shapiro-Wilk test was used to determine whether there was a normal distribution for each outcome. Descriptive statistics (mean and standard deviation or median and interquartile range) were used to present baseline and clinical characteristics. The continuous variables were compared using the T-test (for parametric variables) or the Wilcoxon test (for non-parametric variables) for paired samples to analyze within-group differences before and after intervention. The level of statistical significance was set at p < 0.05 for all analyses. Due to the exploratory nature and small sample size of this feasibility study, statistical analyses were primarily descriptive, and p-values are presented for exploratory purposes only without formal inference. All statistical analyses were conducted in accordance with the study protocol and exploratory objectives. Analyses were performed on participants who completed the intervention. No imputation for missing data was performed, and results are reported based on available data.

## 3. Results

### 3.1. Quantitative results

From October 2024 to January 2025, fifteen people with vestibular disorders were asked to participate in this study. Two of them declined to participate, and thirteen started the virtual training. Finally, a total of ten participants (70% female) completed the intervention (three participants dropped out due to personal reasons, none of the withdrawals were related to adverse events, tolerability issues, or dissatisfaction with the DizzyVR intervention.). The demographic and clinical participants’ characteristics are detailed in Table 4.

**Table 4:** Demographic and clinical characteristics of the participants (n = 10).

Age (median, IQR)	64 (19)
**Gender, n (%)**	
Female	7 (70%)
Male	3 (30%)
**Body Mass Index (mean, SD)**	27.2 (3.9)
**Ethnicity, n (%)**	
Caucasian	9 (90%)
Latin American	1 (10%)
**Diagnosis, n (%)**	
Lindsay-Hemingway Syndrome	2 (20%)
Vestibular migraine	2 (20%)
Cerebellar ataxia	1 (10%)
Meniere’s disease	1 (10%)
Vestibular hypofunction	1 (10%)
BPPV	1 (10%)
Visual vertigo	1 (10%)
SSCDS	1 (10%)
**Type of vestibulopathy, n (%)**	
Peripheral	6 (60%)
Central	3 (30%)
Both	1 (10%)
**Affected side, n (%)**	
Unilateral	3 (30%)
Bilateral	7 (70%)
**Months since diagnosis (median, IQR)**	1 (0.5)
**VR-previous experience, n (%)**	
Yes	1 (10%)
No	9 (90%)

**IQR:** interquartile range, **SD:** standard deviation, **BPPV:** Benign Paroxysmal Positional Vertigo, **SSCDS:** Superior Semicircular Canal Dehiscence Syndrome.

### 3.2. Feasibility

**Fig 8:**
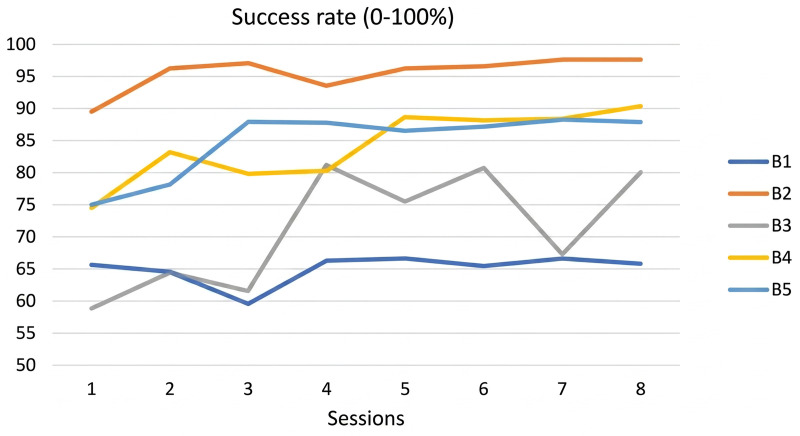
Average success rates (%) across game blocks and training sessions in the DizzyVR program.

Among the 13 participants who initiated the intervention, 10 completed the study, resulting in a completion rate of 76.9%. The session adherence rate among participants who completed the intervention was 91.25%. Five participants completed all eight of the planned training sessions, with an average attendance of 7.3 sessions (SD 0.82). The mean success rates of the games per session were 65.04 ± 2.34% (Block 1), 95.5 ± 2.75% (Block 2), 71.17 ± 9.21% (Block 3), 84.17 ± 5.64% (Block 4), and 84.85 ± 5.20% (Block 5) (see Fig 8). All the participants accomplished the highest level of the games (involving higher speed, a bigger playing area, a smaller scale, and/or a zig-zag direction), except for Block 1, which was considered the most difficult. These results suggest high adherence and engagement with the DizzyVR protocol.

### 3.3. Perceived usability and satisfaction

The mean SUS score was 68.5 (SD 14.29), which corresponds to good usability. Lower scores were mainly associated with item 4, “I think that I would need the support of a technical person to be able to use this system”. The mean USEQ score was 24.6 (SD 4.37), which indicates excellent user satisfaction with the virtual system.

### 3.4. Safety

Three participants (30%) experienced adverse events (nausea, disorientation) during the DizzyVR training, which were resolved in the following sessions. The total number of sessions in which these events were recorded was five (6.84% of the total number of training sessions completed). Simulator Sickness Questionnaire scores decreased from the first to the last training session in the three dimensions (nausea, oculomotor, and disorientation), as well as in the total score (Table 5). These findings indicate that DizzyVR is generally safe and well-tolerated.

**Table 5:** Simulator Sickness Questionnaire scores.

Simulator Sickness Questionnaire dimensions (scores*)				
Nº training session	Nausea *(0-66.78)*	Oculomotor *(0-53.06)*	Disorientation *(0-97.44)*	Total SSQ *(0-812.62)*
*1st session* *8th session*	*28.6 (40.5)* *23.85 (42.93)*	*64.43 (56.85)* *22.7 (34.11)*	*90.48 (97.44)* *55.68 (83.52)*	*669.05 (657.32)* *408.40 (366.67)*

**SSQ:** Simulator Sickness Questionnaire.

*Scores are reported as median (interquartile range-IQR).

### 3.5. Exploratory clinical outcomes

Table 6 shows the comparison between post and pre-intervention for all clinical outcomes. Mean pre–post changes with 95% confidence intervals are also presented to support interpretation of exploratory clinical outcomes. As shown in Table 6, although there was an improvement in dizziness symptoms (DHI), the difference was not statistically significant. In contrast, the TUG test score decreased significantly from 9.53 (SD 3.24) seconds to 7.34 (2.53) seconds, which suggests an exploratory pre–post change in gait speed (p = 0.002). Exploratory pre–post changes were also observed in balance confidence (p = 0.007) and stability during walking (p = 0.012). To avoid overinterpretation in this small feasibility sample, clinical outcomes are reported descriptively, and p-values are presented for exploratory purposes only.

**Table 6:** Outcome measures for participants at baseline and after DizzyVR training.

Outcome measure	Baseline (T0) *1*^*st*^ *session*	End training (T2) *10*^*th*^ *session*	Mean Change (95% CI)	T-Student/Wilcoxon *p-value*
DHI *(0–100)*^ꝉ^	47 ± 26.11	36 ± 26.26	−11.0 (−23.64 to 1.64)	0.081
TUG *(sec)*^ꝉ^	9.53 ± 3.24	7.34 ± 2.53	−2.19 (−3.30 to −1.08)	0.002*
ABC *(0–100)*^ꝉ^	54.52 ± 21.28	66.50 ± 20.69	11.98 (4.20 to 19.75)	0.007*
FGA *(0–30)*^ǂ^	28 (7.5)	30 (0.50)	-------------	0.012*

**ABC:** Activities-specific Balance Confidence, **DHI:** Dizziness Handicap Inventory, **FGA:** Functional Gait Assessment, **sec:** seconds, **TUG:** Timed Up and Go.

ꝉ Mean±SD, ^ǂ^ Median (IQR), * p < 0.05.

### 3.6. Qualitative results

Nine of the 10 patients recruited for the study completed the final semi-structured interview regarding their experiences with the DizzyVR system. The interviews lasted between 4:30 and 13:30 minutes (mean = 7.28 minutes).

#### Usability and satisfaction.

Most of the participants (n = 7) stated that they found the DizzyVR tool easy to use, although two of the patients (P1 and P4) acknowledged that some exercises were more difficult to master than others. When asked whether they felt they could use the device at home without the supervision of a physical therapist, two patients (P8 and P10) felt that they would not be able to use it at home without the supervision of a professional due to fear of possible falls or other symptoms. Likewise, one of the participants acknowledged that it was difficult to use in consultation (P6) and needed a guide to perform the treatment correctly. However, the same participant stated that after completing the sessions, he felt able to carry them out at home.

*“For my part, I think that you need someone to guide you to be able to use it correctly. But as I have been doing them, they have seemed simple and I have not found it difficult to adapt to them, so I think that after the sessions I feel able to use them at home by myself” (Participant 6).*

Almost all participants (n = 8) were satisfied with the results obtained with the DizzyVR tool, reporting perceived improvements in vertiginous symptoms.

Regarding the usefulness of the device, most participants (n = 6) rated its usefulness positively; however, two participants (P1 and P10) commented that although they had improved, they expected better results than those obtained. Only one participant reported not having noticed any change after treatment (P9).

*“I am very satisfied, but I want to keep improving because I am not fully rehabilitated. Getting out of the car and starting to walk still at that moment my symptoms increase, but it is specifically in that situation. So I am happy, but not totally satisfied. That’s why I rate it as 7” (Participant 4).*

Most patients did not suggest any improvement proposals and considered the tool to be complete, easy to use, and confessed to feeling confident. In any case, we would like to highlight as a suggestion for improvement the one provided by P7 in relation to the glasses that are part of this device. This participant reported that the glasses used were too large and uncomfortable. On the other hand, P3 commented that she would add other, more dynamic exercises, and P4 insisted that the treatment with this tool should be economically accessible to all those who needed it.

#### Safety.

Most of the participants (n = 8) stated that they felt safe while performing the exercises. Only P2 reported performing the treatment while seated due to fear of standing. Five of the patients (P1, P4, P6, P8, P10) commented that they had had some kind of vertiginous sensation during the sessions; however, they acknowledged that it was brief and occasional and that in no case did it prevent them from continuing with the development of the session.

*“I felt safe. At no time did I think I was going to fall. It is also true that I had a physiotherapist near me, and I never thought that anything would happen to me. Also, while I was using it, I didn’t feel dizzy. Even on those days when I felt very bad with vertigo, I came here, put on the glasses to do the exercises, and that was the moment when I was fine, and the vertigo disappeared” (Participant 3).*

*“I have previously received other treatments, but this one is less stressful. With the previous treatment, I was dizzy. The side effects are less than in the first treatment. Now I can go out in the street right after the treatment session without having to hold on to someone” (Participant 7).*

Only in the case of P6 were modifications made to the treatment by reducing the duration of certain exercises.

*“It is a system that has surprised me. It is true that in the first sessions I was afraid of it, because I finished regularly and I had to shorten the time to perform some exercises, but I have always been able to finish it. In the last sessions, I have felt good, without any kind of discomfort, and I have been seeing the evolution in me in many things, such as waking up and not getting dizzy in the bathroom, and also not with the computer.” (Participant 6).*

#### Perceived clinical effects.

It should be noted that all the patients agreed that they were not completely recovered, except one participant who commented that she was completely satisfied with her recovery (P8).

*“I have not achieved one hundred percent of the results I was aiming for. It is true that it is too early to say, but I have improved quite a bit. I feel much better than years and months ago” (Participant 3).*

Most of the patients (n = 6) had not received other vestibular rehabilitation treatment before, while three of the participants (P2, P4, and P7) had been treated with other vestibular rehabilitation systems.

*“This is the first time I have received vestibular treatment. For me, it has been very simple, and I think that most people could use this tool. I also think it is quite comprehensive” (Participant 3).*

These three participants stated that treatment with the DizzyVR tool had been less stressful for them, had generated fewer side effects for them, and had been much more motivating to work with this device.

*“I have been treated in different parts of the world, like Japan, Colombia, Hawaii, and Florida. They gave me intratympanic injections, they put me in a hyperbaric chamber, they opened my head, I have a titanium plate...I tell you, it is the million-dollar ear...I have tried everything, and this is the only treatment that has been beneficial for me. That people who suffer from this can have a glimmer of hope is priceless. It has been three very hard years, and with this treatment is when I have seen the light” (Participant 4).*

We also wanted to know whether the participants would recommend the use of this device to other people who might need it and, on the other hand, whether they would use it again if necessary. All participants responded positively to these questions. P9 specified that he would recommend it if he were convinced that it gave good results.

These findings, both quantitative and qualitative, suggest that DizzyVR is a feasible, safe, and well-received intervention for individuals with vestibular disorders, with promising exploratory findings related to functional outcomes and patient experience.

## 4. Discussion

Our findings indicated that DizzyVR is practically applicable, safe, and has very high adherence, acceptable usability and satisfaction ratings, and mild, short-lived adverse events. Such results go together with the 12 studies found in the background, which have equally reported that VR-based vestibular rehabilitation is generally tolerated, motivating, and safe to implement.

Regarding clinical outcomes, our exploratory pre–post changes suggested improvements in gait speed and balance confidence, and gait stability. This is consistent with other previous evidence that also proved improvements of dizziness, gait, and balance with immersive VR-based or task-specific interventions, and the immersive VR may complement conventional vestibular rehabilitation by supporting adherence and functional restoration.

This study evaluated a new virtual reality system, the DizzyVR system. The system appears feasible, usable, acceptable, and safe in patients with vestibular disorders. The secondary purpose of this study was to explore preliminary clinical outcomes associated with the use of this technological system. By designing customizable virtual exercises based on clinical experts’ advice, we observed exploratory pre–post changes in physical ability and participants’ confidence in performing ADL. In other studies, the design of immersive virtual environments was performed in collaboration with Otorhinolaryngology services and the guidelines of the American Physical Therapy Association [27]. That is, the majority of the older methods were based on commercial or generic serious games not explicitly designed to conduct a process of vestibular rehabilitation [25], whereas Le Perf et al. used 360 postural control exercises with a circular optokinetic stimulation and HTC Vive (r) software [27]. Ozdil et al. tested a serious game to test its feasibility with patients in vestibular rehabilitation [15]. DizzyVR accomplishes and aligns precisely with the main exercises described in the Cawthorne-Cooksey blocks.

At the end of the first session, most participants in our study said that the system was easy to use and safe. A few felt dizzy or uncomfortable at the beginning, but this improved over time, and most would recommend DizzyVR. The high SUS and USEQ scores support that the system was well accepted [19,20]. Nehrujee et al. demonstrated that the use of low-cost systems together with virtual environments is useful and safe in the rehabilitation processes in patients with vestibular disorders [28]. The SSQ scores also showed fewer simulator sickness problems over time [21]. These outcomes indicate that the DizzyVR system was well tolerated by the participants. Le Perf et al. proved that the use of VR-based rehabilitation systems was a well-accepted technological system in patients with vestibular disorders [27].

After eight sessions, pre–post improvements in gait speed, balance confidence, and gait stability were observed; however, causality cannot be inferred without a control group in walking and balance. The TUG test times improved, meaning people could stand up, walk, and sit down faster and more steadily. TUG test scores greater than 13.5 seconds have been linked to a higher risk of falls in older adults [23] and in patients with vestibular disorders. In our study, the TUG scores improved after training, which may represent a clinically relevant change for daily functional mobility and safety. The TUG improvement of 2.19 seconds represents a clinically meaningful change (MCID ~1.0–1.5s), as improvements within this range are typically considered significant for patients with vestibular disorders or older adults, meaning they can notice the difference in their daily activities.

Participants also scored higher on the FGA. Because fall risk is associated with scores ≤22 [25], our participants were already at relatively low risk at baseline. This suggests that the observed gains, though statistically significant, may reflect a ceiling effect, limiting the ability of the FGA to capture further functional improvements in this sample.

Balance confidence improved, too. ABC scores below 67 suggest a higher fall risk in older adults [24]. Our participants’ average ABC score increased, meaning they felt more confident doing ADL without fear of losing balance.

Whereas the change in DHI scores was not sufficiently large to reach statistical significance, it was found that participants reported reduced dizziness symptoms after training. The values of baseline DHI showed moderate disability, which is a positive trend [22]. Other results need to be interpreted carefully: FGA scores were already close to the maximum at baseline, and this shows the absence of risk of falls, and the ceiling effect may have constrained the possibility of further increase. Similarly, the decrease in TUG could be partly due to learning effects rather than a true functional change. We think that these outcomes are due to the selection of the participants in our study; there was heterogeneity in the diagnosis. Another point based on our results is that in the procedure, the sessions were short, and finally, there was a high variability.

Irrespective of these shortcomings, the novelty of our solution is its customizable, block-based architecture directly projected onto the Cawthorne-Cooksey protocol, inclusion of objective kinematic data, and specified progression rules, which, altogether, could be more task-specific, measurable, and motivating rehabilitation than previous VR systems.

These findings align with previous research reporting positive exploratory outcomes associated with VR-based interventions in improving balance and reducing fall risk in similar populations. Ozdil et al. [15] found that 3D virtual reality training improved balance and gait in patients with BPPV. Bonaventurová et al. [14] showed that VR training helped people recover faster after vestibular schwannoma surgery. However, their studies had limits such as small sample sizes, single centers, and short follow-ups, similar to our study’s limits. Unlike their systems, our DizzyVR program uses ADL tasks and blocks that train gaze, postural control, eye-hand coordination, gait, and visual challenges, making it more applicable to real-world rehabilitation contexts.

### 4.1. Limitations

This study has some important limitations. First, the sample size was small, with only ten participants, so the results should be interpreted with caution. Second, there was no control group, which means we cannot say for sure that the improvements were only due to DizzyVR and not to other factors like natural recovery or possible Hawthorne effects.

Third, the duration of the intervention was short, with only eight sessions. This showed positive short-term effects, but we do not know if these improvements will last over time. Fourth, although we used validated questionnaires, some measures relied on what participants reported themselves, which can be subjective.

Fifth, our group included participants with both peripheral and central vestibulopathies, introducing heterogeneity that may limit the interpretation of mechanism-specific effects and how well the results apply to each specific condition. In the future, we need to analyze whether the DizzyVR system can enrich the outcomes in a specific population, such as peripheral or central vestibulopathy.

Finally, there was no blinding of participants or assessors, which could have influenced the results. Having the same therapist involved in recruiting, treating, and assessing participants also introduces a potential risk of bias. Additionally, the novelty of the technology may have enhanced participants’ motivation beyond its actual therapeutic value. The one-week interval between sessions may also have been suboptimal for promoting neuroplasticity compared to more frequent training.

Differences between the planned protocol and the final implementation are acknowledged, including the reduced sample size and minor variations in eligibility criteria, which are inherent to feasibility studies. In future studies, we plan to implement blinding of both therapists and participants and adjust session frequency to minimize these sources of bias and enhance treatment effectiveness.

Data Availability Statement: All relevant data supporting the findings of this study are included within the manuscript.

## 5. Conclusions

This study offers promising preliminary evidence for the feasibility, usability, satisfaction, and safety of the DizzyVR system as a complementary tool for people with vestibular disorders. Our results showed exploratory pre–post changes in balance, gait, and balance confidence, together with good acceptability and minimal side effects.

These findings suggest that DizzyVR can be a useful system to complement traditional vestibular rehabilitation, supporting functional performance and patient engagement.

Future research should include randomized controlled trials with larger and more homogeneous samples, assess long-term retention of benefits, and explore the system’s applicability across different types of vestibular disorders.
